# The Military Cross for an Army Doctor

**Published:** 1915-04-10

**Authors:** 


### The Military Cross for an Army Doctor.

Among those officers on whom the King has been pleased to confer the Military Cross in recognition of gallantry and devotion to duty while serving with the Expeditionary Force is Lieutenant T. W. Clarke, M.B., Royal Army Medical Corps, Special Reserve.

For conspicuous gallantry and great devotion to duty during the past six months. On March 5, at Neuve Eglise, when the 14th Field Ambulance dressing station was destroyed by shell fire (one officer and five men being killed and nineteen wounded therein), Lieutenant Clarke continued to attend on the wounded with great gallantry until he collapsed from his own wound—which he received from the first shell.

